# Dialysis on the Central Venous Catheter at the Time of Arteriovenous Fistula Creation Is Associated with Long-Term Vascular Access Failure

**DOI:** 10.3390/jcm15103819

**Published:** 2026-05-15

**Authors:** Eliza Russu, Réka Bartus, Elena Florea, Alexandru Mureșan, Paula Bândea, Paul Mateica, Ionela Georgiana Tofana, Constantin Claudiu Ciucanu, Eliza-Mihaela Arbănași, Alexandru Petru Ion, Paula Chirilă, Ioan Hosu, Mirela Liana Gliga, Adrian Vasile Mureșan, Emil-Marian Arbănași

**Affiliations:** 1 Department of Vascular Surgery, George Emil Palade University of Medicine, Pharmacy, Science and Technology of Targu Mures, 540142 Targu Mures, Romania; eliza.russu@umfst.ro (E.R.); claudiu.ciucanu@umfst.ro (C.C.C.); adrian.muresan@umfst.ro (A.V.M.); emil.arbanasi@umfst.ro (E.-M.A.); 2 Clinic of Vascular Surgery, Mures County Emergency Hospital, 540136 Targu Mures, Romania; alex.muresan99@yahoo.ro (A.M.); paula_bandea@yahoo.com (P.B.); paulmateica@yahoo.com (P.M.); bodiu_ionela@yahoo.com (I.G.T.); 3 Doctoral School of Medicine and Pharmacy, George Emil Palade University of Medicine, Pharmacy, Science and Technology of Targu Mures, 540142 Targu Mures, Romania; dr.florea.elena@gmail.com (E.F.); arbanasi.eliza@gmail.com (E.-M.A.); petru.ion@umfst.ro (A.P.I.); paulachirila@yahoo.com (P.C.); 4 Regenerative Medicine Laboratory, Centre for Advanced Medical and Pharmaceutical Research (CCAMF), George Emil Palade University of Medicine, Pharmacy, Science and Technology of Targu Mures, 540142 Targu Mures, Romania; 5 Department of Internal Medicine-Nephrology, George Emil Palade University of Medicine, Pharmacy, Science, and Technology of Targu Mures, 540142 Targu Mures, Romania; ioan.hosu@umfst.ro (I.H.); mirela.gliga@umfst.ro (M.L.G.); 6 Department of Nephrology, Mures Clinical County Hospital, 540139 Targu Mures, Romania; 7 Diaverum Dialysis Center, 540139 Targu Mures, Romania

**Keywords:** vascular access, central venous catheter, arteriovenous fistula, dialysis, vascular surgery

## Abstract

**Background/Objectives**: The autologous arteriovenous fistula (AVF) is the primary vascular access (VA) method for hemodialysis (HD), with superior patency and a lower complication rate than arteriovenous graft (AVG) or central venous catheter (CVC). The primary objective of this study is to analyze the impact of dialysis on the CVC at the time of AVF creation on its long-term primary patency. **Methods**: This retrospective, single-center, observational study included 248 patients admitted for AVF creation. Demographic characteristics, comorbid conditions, and laboratory parameters were retrieved from the hospital’s electronic medical records. The primary outcome of the study was long-term AVF failure, defined as the inability to perform hemodialysis through the newly created AVF. **Results**: A total of 132 (53.2%) were receiving dialysis via a CVC at the time of AVF creation. During a mean follow-up of 2.21 ± 1.54 years, 47 patients (18.95%) failed to achieve functional maturation at 6 weeks, and 81 patients (32.66%) developed long-term AVF failure. Demographic characteristics were similar between patients with and without CVC at AVF creation, with no significant differences in age (*p* = 0.358) or sex (*p* = 0.574). Comorbidities and risk factors were also similarly distributed, showing no significant variation. The types of AVF varied by CVC status, with fewer RC-AVF (*p* = 0.038) and more BC-AVF (*p* = 0.032) in patients with a CVC. Failure was significantly more frequent in patients with CVC use at the time of AVF creation than in those without CVC (*p* < 0.001). Kaplan–Meier analysis demonstrated lower long-term patency in patients dialyzed via CVC (*p* < 0.001). In Cox regression analysis, dialysis via CVC at AVF creation was associated with AVF failure (HR: 2.53, *p* < 0.001), and the association remained significant after full adjustment (HR: 3.13, *p* < 0.001). Female sex, active smoking, smaller arterial and venous diameters, and RC-AVF were additional risk factors associated with failure. **Conclusions**: Dialysis via a CVC at the time of AVF creation was associated with an increased risk of long-term AVF failure, even after full adjustment models.

## 1. Introduction

The autologous arteriovenous fistula (AVF) serves as the primary vascular access (VA) method for hemodialysis (HD), exhibiting superior patency and a lower incidence of complications compared with arteriovenous graft (AVG) or central venous catheter (CVC) [1]. In accordance with the guidelines established by the European Society of Vascular Surgery (ESVS), it is recommended to construct an AVF three to six months before the anticipated commencement of HD [1]. However, the performance of AVF over the medium and long term is deemed unsatisfactory, as evidenced by a primary unassisted patency rate of 64% at one year, a primary assisted patency rate of 73%, and a secondary patency rate of 79%, necessitating numerous corrective interventions [1,2,3,4,5]. Moreover, patients with kidney failure exhibit a higher rate of AVF maturation compared to those with chronic kidney disease (CKD) at three months postoperatively [4].

A multitude of factors contribute to AVF failure, including elevated systemic inflammation [6,7,8], neovascularization [8], pathological characteristics of the venous wall [8,9], and preoperative vascular mapping [10]. However, another risk factor is performing dialysis on CVC at the time of surgical AVF creation [5,11]. Thus, Pfister et al. [5] found that patients undergoing hemodialysis during AVF creation had lower primary and secondary patency rates (*p* = 0.037 and *p* = 0.024, respectively). Similarly, Ravani et al. [11] observed an increased incidence of primary patency failure in patients who utilized a CVC at the initiation of hemodialysis (*p* = 0.0005). Conversely, a recent study conducted by Torreggiani et al. [12] documented a higher risk of AVF failure compared with CVC complications (*p* < 0.01) while revealing no significant difference in survival outcomes between the two types of VA.

The primary objective of this study is to analyze the impact of dialysis on the CVC at the time of AVF creation in relation to its long-term primary patency. Furthermore, this study will examine the risk factors associated with the long-term failure of AVF.

## 2. Materials and Methods

### 2.1. Study Design

The present retrospective, single-center observational study includes all patients admitted to the Vascular Surgery Department of the Emergency Clinical County Hospital in Târgu Mureș, Romania, between 2019 and 2024 who underwent an operation to create an upper extremity AVF and subsequently initiated HD using the newly created AVF. Patients whose postoperative AVF demonstrated no immediate thrill or patency, those in whom the AVF was never used for hemodialysis, patients who died before AVF utilization, and patients who underwent kidney transplantation prior to AVF cannulation were excluded from the analysis. Patients with incomplete clinical data in the electronic hospital records and those who received an AVG were also excluded. Eligible patients were categorized first according to the presence or absence of a CVC at the time of AVF creation and subsequently according to the occurrence of long-term AVF failure.

### 2.2. Data Collection

Demographic characteristics, comorbid conditions, and laboratory data were extracted from the hospital’s electronic medical records. The comorbidities recorded included hypertension, atrial fibrillation, ischemic heart disease, prior myocardial infarction, diabetes mellitus, prior stroke, obesity, and active smoking status. Preoperative laboratory data were collected for the following variables: hemoglobin, hematocrit, serum potassium and sodium, creatinine, blood urea nitrogen (BUN), neutrophil, lymphocyte, and monocyte counts, as well as platelet count and serum glucose. In addition, preoperative vascular mapping data were reviewed, and the diameters of the radial artery (RA), brachial artery (BA), basilic vein (BV), and cephalic vein (CV) were recorded when available.

The current study was approved by the Medical Ethics Committee for the Clinical Study of the Drug within the framework of the Târgu Mureș County Emergency Clinical Hospital, Romania, decision no. 4526; approval date: 4 March 2025.

### 2.3. AVF Creation

Patients included in this study underwent radial–cephalic (RC-AVF), brachial–cephalic (BC-AVF), or two-stage brachial–basilic (BB-AVF) fistula creation. The same vascular surgery team performed all procedures. For RC-AVF and BC-AVF, end-to-side (vein-to-artery) anastomosis was performed using a continuous 6-0 suture technique. Following anastomosis, the presence of a palpable thrill along the vein was verified, after which the subcutaneous tissue and skin were closed in layers. For two-stage BB-AVF procedures, the first stage involved creating an anastomosis between the BA and BV, as in RC-AVF and BC-AVF. Vein superficialization was performed as a second-stage procedure 2–3 weeks later, following confirmation of adequate arterialization of the venous outflow segment.

### 2.4. Follow-Up and Outcomes

The primary outcome of the study was long-term AVF failure, defined as the permanent inability to use the newly created AVF for effective hemodialysis due to an access-related cause, including thrombosis, juxta-anastomotic or outflow stenosis non-amenable to or refractory to endovascular salvage, aneurysmal degeneration requiring abandonment, or non-maturation precluding successful cannulation despite a documented attempt at cannulation. Events not attributable to the access itself—namely, patient death from any cause with a functioning AVF, kidney transplantation, switch to peritoneal dialysis, transfer to another center with loss of follow-up, or elective non-use of a patent and mature AVF—were not classified as AVF failure and were instead treated as censoring events in the time-to-event analyses. Moreover, the 6-week maturation failure was defined according to the ESVS 2018 vascular access guidelines as failure of the AVF to reach a venous diameter ≥ 6 mm, a venous depth ≤ 6 mm, and an access flow ≥ 600 mL/min at six weeks postoperatively [1]. The two endpoints were treated hierarchically: a 6-week maturation failure was counted as a long-term AVF failure event only if the access ultimately could not be used for HD. Conversely, AVFs that failed to mature at 6 weeks but were subsequently used for HD were classified as 6-week maturation failures and as long-term functional AVFs in the survival analysis. Patient follow-up was conducted through a comprehensive review of hospital medical records and was further corroborated by direct communication with chronic dialysis centers. Data were collected for all patients until AVF failure occurred or until 31 May 2025, whichever came first. The patients were monitored following discharge for an average period of 2.21 ± 1.54 years.

### 2.5. Statistical Analysis

All statistical analyses were performed using SPSS for macOS, version 29.0.2.0 (SPSS Inc., Chicago, IL, USA). Normally distributed variables (age, follow-up duration, and arterial and venous diameters) are expressed as mean ± standard deviation, whereas non-normally distributed variables (laboratory parameters) are reported as median and interquartile range (Q1–Q3). Between-group comparisons were performed using the independent-samples Student’s *t*-test for normally distributed variables and the Mann–Whitney U test for non-normally distributed variables. Categorical variables, presented as absolute frequencies and percentages, were compared using the chi-square test. Kaplan–Meier survival analyses were used to evaluate the association between hemodialysis via a CVC at the time of AVF creation and long-term AVF failure in the overall cohort and in the RC-AVF and BC-AVF subgroups. Survival curves were compared using the log-rank test. Cox proportional hazards regression analyses were performed to determine the predictive value of variables of interest for long-term AVF failure. Furthermore, four distinct adjustment models were employed to evaluate the impact of hemodialysis on the CVC at the time of AVF creation and long-term AVF failure: Model 1 was unadjusted; Model 2 was adjusted for age and sex; Model 3 was further adjusted for cardiovascular risk factors (diabetes mellitus, hypertension, ischemic heart disease, and active smoking) and renal function (BUN, creatinine); and Model 4 additionally included AVF type and preoperative vascular mapping parameters. All tests were two-sided, and *p*-values less than 0.05 were considered statistically significant.

## 3. Results

A total of 248 patients were analyzed in the current study, with a mean age of 62.97 ± 13.91 years. The study population comprised 150 males (60.48%) and 98 females (39.52%). Hypertension constituted the predominant cardiovascular comorbidity, affecting 224 patients (90.32%). Ischemic heart disease was identified in 109 patients (43.95%), whereas both atrial fibrillation and prior myocardial infarction were each reported in 23 patients (9.27%). A previous cerebrovascular event was documented in 12 patients (4.84%). Diabetes represented a frequent metabolic comorbidity and was present in 91 individuals (36.69%). In terms of risk factors, active smoking was reported in 46 patients (18.55%), while obesity was observed in 26 patients (10.48%). Regarding AVF configuration, RC-AVF was the most commonly constructed access type in 128 patients (51.61%), followed by BC-AVF in 90 patients (36.29%) and BB-AVF in 30 patients (12.10%). Preoperative vascular mapping was available for 212 patients, with mean arterial and venous diameters of 3.18 ± 1.17 mm and 3.21 ± 0.93 mm, respectively. The majority of AVFs were created in the non-dominant upper extremity (83.87%), and 139 procedures (56.05%) were performed in an outpatient setting. At the time of AVF formation, 132 patients (53.22%) were receiving hemodialysis via a central venous catheter, while the remaining patients were predialysis candidates. During follow-up, failure to achieve functional maturation at 6 weeks occurred in 47 patients (18.95%), and long-term AVF failure was documented in 81 patients (32.66%) (Table 1).

Demographic characteristics were similar between patients with and without CVC at AVF creation, with no significant differences in age (*p* = 0.358) or sex (*p* = 0.574). Comorbidities and risk factors were also similarly distributed, showing no significant variation. However, baseline laboratory data revealed higher serum creatinine (*p* = 0.023) and BUN (*p* < 0.001) levels in patients with a CVC at the time of AVF creation, along with lower neutrophil counts (*p* = 0.048). The types of AVF varied by CVC status, with fewer RC-AVF (*p* = 0.038) and more BC-AVF (*p* = 0.032) in patients with a CVC. Although the 6-week maturation failure rate was similar (*p* = 0.196), long-term AVF failure was significantly higher in patients with a CVC at the time of AVF creation (*p* < 0.001), indicating that CVC presence is linked to poorer long-term access outcomes despite comparable baseline profiles (Table 1).

Patients who experienced long-term AVF failure were disproportionately female (*p* = 0.006), whereas no differences were observed with respect to age (*p* = 0.775) or body mass index (*p* = 0.270). This group demonstrated a higher prevalence of risk factors, including active smoking (*p* = 0.015) and obesity (*p* = 0.046), while the distribution of major cardiovascular comorbidities and baseline laboratory indices remained broadly comparable across groups. Preoperative vascular mapping identified lower arterial (*p* = 0.005) and venous (*p* = 0.005) diameters among individuals with long-term AVF failure, suggesting an anatomic substrate predisposing to adverse outcomes. Regarding access configuration, RC-AVF was more frequently constructed in this cohort (*p* = 0.013). In contrast, BB-AVF was less commonly employed (*p* = 0.046), and procedures were less often performed in the outpatient setting (*p* = 0.033). During follow-up, these patients experienced a significantly higher rate of 6-week maturation failure (*p* < 0.001) and were more likely to undergo dialysis via a CVC at the time of AVF creation (*p* < 0.001) (Table 2). Overall, these results highlight the complex interplay between sex, modifiable behavioral factors, vascular size, and access type in influencing the long-term durability of AVFs.

**Figure 1:**
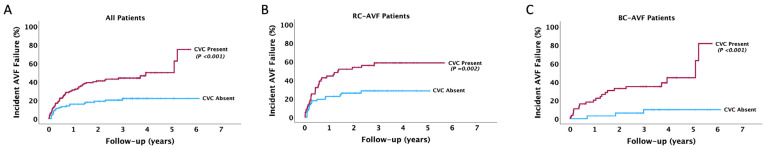
Kaplan–Meier curves for long-term AVF failure according to the presence of a CVC status at AVF creation, in (**A**) the entire cohort of patients, (**B**) RC-AVF patients, and (**C**) BC-AVF patients. *p*-values reflect log-rank testing for between-group differences.

In the Kaplan–Meier survival curve, dialysis on CVC at the time of AVF creation was associated with a higher long-term AVF failure (*p* < 0.001) in the entire cohort, as well as in the RC-AVF (*p* = 0.002) and BC-AVF (*p* < 0.001) subgroups (Figure 1).

In Cox regression analysis, female sex (HR: 1.92, *p* = 0.003), active smoking (HR: 1.89, *p* = 0.011), and creation of an RC-AVF (HR: 1.98, *p* = 0.003) were independently associated with an increased risk of long-term AVF failure. Conversely, the BB-AVF configuration was associated with a lower risk of failure (HR: 0.61, *p* = 0.043), as well as larger preoperative arterial (HR: 0.73, *p* = 0.029) and venous (HR: 0.68, *p* = 0.005) diameters, which emerged as significant protective factors (Table 3).

Dialysis via a CVC at the time of AVF creation was strongly associated with subsequent AVF failure (HR: 2.53, *p* < 0.001) (Table 4). Sequential multivariable adjustment did not attenuate this association. After adjustment for age and sex, the risk remained significantly elevated (HR: 2.86, *p* < 0.001), and inclusion of major cardiovascular risk factors (diabetes, hypertension, ischemic heart disease, and active smoking) yielded similar results (HR: 2.88, *p* < 0.001). In the fully adjusted model that additionally accounted for AVF type and preoperative vascular mapping, the association was even greater (HR: 3.13, *p* < 0.001) (Table 4). This analysis confirmed that the association between CVC use at the time of AVF creation and AVF failure persisted despite comprehensive adjustment for demographic, clinical, anatomical, and procedural factors. Collectively, these findings demonstrate a robust and independent relationship between CVC dialysis at AVF creation and long-term AVF failure risk.

## 4. Discussion

The main result of the current study is that dialysis via a CVC at the time of AVF creation was strongly associated with an increased risk of long-term primary patency failure of the AVF. The association persisted even after adjustment for age, sex, comorbidities, vascular anatomy, and AVF type, indicating that it is independent of these factors. Moreover, female sex, active smoking, smaller arterial and venous diameters, and RC-AVF were additional predictors of long-term failure. These findings underscore the negative impact of catheter-dependent dialysis on AVF construction and the importance of vascular anatomy and modifiable risk factors in long-term access patency.

Similar to the results from the current study, female sex was associated with a higher risk of maturation and long-term AVF failure [13,14,15,16,17,18,19]. In the study by Hoffstaetter et al. [13] on 1077 patients with AVF, women were found to require a more proximal AVF at a higher level, with a lower maturation rate. In previous studies published by our team, we observed that the female gender was associated with long-term AVF failure in RC-AVF patients [14,15]. This phenomenon can be attributed to different patterns of inflammation, hormonal signaling, immune-cell activity, and extracellular matrix remodeling observed in females, as explained by Chan et al. [16]. Furthermore, Kudze et al. [17], using a murine model, showed that although early venous dilation and wall thickening are similar, females exhibit lower AVF patency, which is linked to decreased shear stress, reduced laminar flow, and an inability to increase the levels of key flow-responsive mediators. Preoperative vascular mapping provides objective measurements and guides site selection for AVF creation, as outlined in the ESVS guidelines [1]. However, several studies demonstrated that beyond diameter thresholds, the vascular mapping contributes to AVF outcomes by characterizing venous distensibility, arterial flow, and peak systolic velocities, parameters repeatedly linked with early failure and non-maturation in observational studies and meta-analyses [8,14,20,21,22,23,24,25].

The ESVS guidelines [1] state that among AVF types, RC-AVF demonstrates the weakest long-term performance when compared with BC-AVF and BB-AVF. However, evidence in the literature is inconsistent. Some studies report that RC-AVF outperforms more proximal AVFs in both short- and long-term outcomes [5,26], whereas other investigations find no significant differences between AVF types [7,27]. In a meta-analysis of 15 studies, McGrogan et al. [28] concluded that RC-AVF is associated with worse primary and secondary patency than BC-AVF in older adults. Similarly, Kim et al. [29] found that BC-AVF provides superior primary patency and necessitates fewer interventions in octogenarian patients. According to our study, RC-AVF was a risk factor for long-term AVF failure (HR: 1.98, *p* = 0.003). Clinically, access planning should balance patency outcomes with the preservation of future access sites: while BC-AVF may provide better patency in the elderly, patients have limited options for AVF creation, so an AVF should be constructed as distally as possible whenever feasible, with individualized decision-making based on anatomy, comorbidities, and life expectancy.

Prior use of dialysis CVCs has been consistently associated with inferior long-term AVF outcomes. Proposed mechanisms include central venous stenosis, endothelial injury, thrombosis, and chronic inflammation, all of which impair venous outflow and the vascular remodeling required for fistula maturation [30,31]. In a retrospective analysis, Diep et al. [30] demonstrated that prior use of tunneled catheter dialysis (TDC) was associated with reduced AVF performance, with the effect more pronounced when the catheter was placed ipsilaterally rather than contralaterally. Likewise, Wongmahisorn et al. [31] observed lower maturation rates in newly created AVFs among patients with prior non-tunneled CVCs. In contrast, the systematic review and meta-analysis by Koudounas et al. [32] reported no significant effect of tunneled dialysis catheters on AVF functional maturation and failure rates. Similarly, Kim et al. [33] did not find any association between TDC sidedness and AVF maturation or early failure in a cohort of 187 patients.

Beyond the clinical-epidemiological association described above, the strong, dose-independent relationship between catheter-dependent dialysis at the time of AVF creation and long-term access failure observed in our cohort invites a more explicitly mechanistic interpretation. Vazquez-Padron et al. [34] recently proposed a revised paradigm of AVF failure centered on the acute biomechanical response to fistula creation and the ensuing remodeling of the venous wall under supraphysiological hemodynamic conditions. Within this framework, early remodeling is characterized by depletion of vascular smooth muscle cells and prominent inflammatory infiltration, while later stages are dominated by extracellular matrix-producing myofibroblasts and fibroblasts [35]. Leveraging single-cell RNA sequencing of venous wall specimens, Martinez et al. [35] further demonstrated that failed AVFs exhibit persistent inflammation alongside an exaggerated wound-healing response—findings that provide a plausible biological substrate through which a pre-existing pro-inflammatory milieu could compromise fistula maturation. A parallel line of evidence implicates central venous catheters themselves as drivers of this maladaptive vascular phenotype. Wang et al. [36] reported substantial variability in surface roughness across commercially available CVCs, with coefficients of friction—at both catheter tips and bodies—correlating positively with surface roughness, thereby providing a mechanical rationale for the development of central vein stenosis (CVS), which is thought to originate from endothelial injury inflicted during venous cannulation and perpetuated by the chronic presence of an indwelling foreign body [37]. Catheter micromotion driven by respiration and head and postural movements, together with the increased flow, turbulence, and altered shear stress that accompany AVF maturation, promotes platelet deposition and progressive venous wall remodeling [37]. The presence of CVS introduces a biomechanical mismatch between the compliant venous segment used for AVF creation and the stenotic, less compliant central outflow tract, thereby impairing the adaptive remodeling process required for successful maturation. Taken together, these observations suggest that catheter-dependent patients enter AVF creation with a venous bed already primed by endothelial injury, chronic inflammation, and incipient fibrotic remodeling—a substrate that may predispose to the unfavorable long-term outcomes documented in our analysis.

Clinically, these findings endorse strategies that reduce catheter dependence by emphasizing early nephrology consultation, prompt AVF planning, and predialysis AVF creation when possible. Risk assessment should consider female sex, active smoking, small arterial and venous sizes, and RC-AVF configuration as indicators of lower long-term patency. This warrants personalized access choices, careful preoperative vascular mapping, and proactive modification of behavioral risk factors. Overall, these insights call for a shift toward early access planning and minimizing catheter use to improve long-term vascular access outcomes for hemodialysis patients.

### 4.1. Future Direction: Integrating Computational Hemodynamic Modeling

Although vessel diameter remains the cornerstone of ESVS-recommended preoperative vascular mapping [1] and is consistently associated with maturation and patency in observational cohorts [10,14,20,21,22,23,24,25], it cannot capture the disturbed-flow patterns, wall shear stress (WSS) gradients, and oscillatory shear index (OSI) values that drive endothelial mechanotransduction, neointimal hyperplasia, and stenosis at the juxta-anastomotic region. Several of these parameters are inherently difficult to measure directly in the clinical setting because they require high-resolution, time-resolved imaging combined with patient-specific vessel reconstruction. Emerging computational approaches are progressively bridging this gap. Patient-specific computational fluid dynamics (CFD) models, built from preoperative duplex ultrasound, magnetic resonance, or computed tomography angiography data, can simulate post-anastomotic flow distributions, predict postoperative brachial-artery flow volumes for different surgical configurations, and identify regions of pathological shear stress prone to subsequent stenosis [38,39,40,41,42]. More recently, the comprehensive review by Nowacki et al. [41] synthesized the current state of computational AVF research, highlighting how CFD has consistently linked low WSS and high OSI to regions susceptible to neointimal hyperplasia and how machine learning (ML) models are increasingly able to predict maturation, stenosis, and access failure with promising accuracy.

### 4.2. Study Limitations

The current study has several limitations that should be mentioned and addressed in future studies. First, its retrospective, single-center design limits causal inference and generalizability to other populations and clinical practice. Second, because of the retrospective, single-center design and the fact that a substantial proportion of patients had their CVC placed at external dialysis units or referring hospitals before admission for AVF creation, the precise interval between CVC placement and AVF creation—and therefore the cumulative catheter dwell time prior to AVF utilization—could not be reliably reconstructed for the majority of patients in the CVC subgroup. Third, preoperative vascular mapping data were unavailable for all patients, potentially introducing selection bias into analyses that included vessel diameters. Additionally, information on the type and site of the CVC, as well as catheter dwell time and infection episodes, which may influence long-term patency, could not be fully evaluated. Furthermore, the anatomical relationship between the CVC insertion site and the subsequently created AVF could not be systematically evaluated because catheter laterality and insertion site documentation were incomplete in retrospective records. Ipsilateral catheter placement may contribute to central venous stenosis, venous hypertension, and impaired venous outflow, particularly affecting the maturation and patency of proximal AVFs. Finally, because postoperative follow-up was conducted by the affiliated nephrology departments rather than by our vascular surgery service, quantitative hemodynamic data on the maturing AVF—including access flow, peak systolic velocities, and venous distensibility—were not systematically recorded in the hospital’s electronic database. This precluded direct correlation between hemodynamic variables and long-term failure. As discussed above, future work from our group will specifically address this limitation by combining prospective hemodynamic monitoring with patient-specific computational modeling.

## 5. Conclusions

In this retrospective cohort of patients undergoing AVF creation, dialysis via a CVC at the time of surgery was independently and strongly associated with an increased risk of long-term AVF failure, even after full adjustment models for demographic, clinical, anatomical, and procedural factors. Female sex, active smoking, smaller preoperative arterial and venous diameters, and RC-AVF configuration further increased failure risk, whereas BC-AVF demonstrated a protective effect. These findings emphasize the clinical importance of avoiding prolonged catheter dependence whenever feasible, optimizing vessel selection, and addressing modifiable risk factors to improve long-term vascular access outcomes. Strategies promoting earlier referral and timely AVF planning may reduce the need for catheter use at creation and enhance patency.

## Figures and Tables

**Table 1:** Comparison of baseline demographic, clinical, laboratory, and AVF-related characteristics between patients with and without a CVC at the time of AVF creation.

Variables	All Patients n = 248	CVC Absent n = 116	CVC Present n = 132	*p* Value
Age, mean ± SD	62.97 ± 13.91	63.84 ± 14.22	62.21 ± 13.63	0.358
Male, no. (%) Female, no. (%)	150 (60.48%) 98 (39.52%)	68 (58.62%) 48 (41.38%)	82 (62.12%) 50 (37.88%)	0.574
BMI, no. (%)	28.02 ± 6.51	29.15 ± 6.83	27.35 ± 6.27	0.156
Comorbidities and Risk factors, no. (%)				
Hypertension	224 (90.32%)	103 (88.79%)	121 (91.67%)	0.445
Atrial Fibrillation	23 (9.27%)	8 (6.90%)	15 (11.36%)	0.226
Diabetes	91 (36.69%)	42 (36.21%)	49 (37.21%)	0.882
Ischemic Heart Disease	109 (43.95%)	49 (42.24%)	60 (45.45%)	0.611
History of Myocardial Infarction	23 (9.27%)	9 (7.76%)	14 (10.61%)	0.453
History of Stroke	12 (4.84%)	6 (5.17%)	6 (4.55%)	0.818
Active Smoking	46 (18.55%)	17 (14.66%)	29 (21.97%)	0.139
Obesity	26 (10.48%)	11 (9.48%)	15 (11.36%)	0.629
Laboratory data, median (Q1–Q3)				
WBC	7.64 (6.13–9.44)	7.88 (6.50–9.61)	7.27 (6.01–9.34)	0.145
Potassium mmol/L	5.10 (4.57–5.62)	5.09 (4.51–5.46)	5.16 (4.65–5.73)	0.066
Sodium mmol/L	139 (137–141)	139.5 (138–141)	138.05 (137–140.25)	0.053
Glucose (mg/dL)	103 (89.0–137.4)	97.55 (88.6–127.12)	105.7 (91.0–160.0)	0.073
BUN (mg/dL)	126.6 (92.0–163.3)	112.4 (85.6–146.7)	145.4 (108.51–173.83)	<0.001
Creatinine (mg/dL)	6.37 (5.05–8.06)	6.12 (4.86–7.76)	6.77 (5.28–8.33)	0.023
Hemoglobin g/dL	10.0 (8.58–11.4)	10.5 (8.59–11.4)	9.8 (8.53–11.37)	0.327
Hematocrit %	30.6 (26.7–37.08)	30.9 (26.74–35.02)	30.35 (26.7–35.0)	0.649
Neutrophils × 10^3^/μL	5.27 (4.05–6.62)	5.65 (4.31–6.98)	4.81 (3.76–6.43)	0.048
Lymphocytes × 10^3^/μL	1.41 (1.05–1.97)	1.41 (1.14–1.97)	1.41 (1.02–1.95)	0.444
Monocyte × 10^3^/μL	0.61 (0.47–0.78)	0.59 (0.45–0.77)	0.65 (0.51–0.79)	0.247
PLT × 10^3^/μL	217 (173.5–282)	233 (182.5–289)	205 (170–275.25)	0.052
Vascular Mapping Determinations *, median (Q1–Q3)				
Arterial Diameter (mm)	3.18 ± 1.17	3.05 ± 0.93	3.29 ± 1.34	0.453
Vein Diameter (mm)	3.21 ± 0.93	3.07 ± 0.74	3.33 ± 1.06	0.104
AVF Type and Placement, no. (%)				
RC-AVF	128 (51.61%)	68 (58.62%)	60 (45.45%)	0.038
BC-AVF	90 (36.29%)	34 (29.31%)	56 (42.42%)	0.032
BB-AVF	30 (12.10%)	14 (12.07%)	16 (12.12%)	0.990
Non-Dominant Upper Limb	208 (83.87%)	103 (88.79%)	105 (79.55%)	0.082
Outpatients, no. (%)	139 (56.05%)	68 (58.62%)	71 (53.79%)	0.051
6-week Maturation Failure, no. (%)	47 (18.95%)	18 (15.52%)	29 (21.97%)	0.196
AVF Failure, no. (%)	81 (32.66%)	23 (19.83%)	58 (43.94%)	<0.001
Follow-up period (years) mean ± SD	2.21 ± 1.54	2.42 ± 1.46	2.02 ± 1.58	0.046

* The value of preoperative vascular mapping determinations is available only for a group of 212 patients from the entire cohort.

**Table 2:** Comparison of baseline demographic, clinical, laboratory, and AVF-related characteristics between patients with and without long-term AVF failure.

Variables	Functional AVF n = 167	AVF Failure n = 81	*p* Value
Age, mean ± SD	62.81 ± 14.54	63.32 ± 12.59	0.775
Male, no. (%) Female, no. (%)	111 (66.47%) 56 (33.53%)	39 (48.15%) 42 (51.85%)	0.006
BMI, no. (%)	27.48 ± 6.54	28.64 ± 6.48	0.270
Comorbidities and Risk factors, no. (%)			
Hypertension	103 (88.79%)	121 (91.67%)	0.445
Atrial Fibrillation	14 (8.38%)	9 (11.11%)	0.487
Diabetes	58 (34.73%)	33 (40.74%)	0.357
Ischemic Heart Disease	74 (44.31%)	35 (43.21%)	0.870
History of Myocardial Infarction	13 (7.78%)	10 (12.35%)	0.252
History of Stroke	9 (5.39%)	3 (3.70%)	0.562
Active Smoking	24 (14.37%)	22 (27.16%)	0.015
Obesity	13 (7.78%)	13 (16.05%)	0.046
Laboratory data, median (Q1–Q3)			
WBC	7.4 (6.02–9.36)	8.09 (6.98–9.46)	0.143
Potassium mmol/L	5.09 (4.54–5.58)	5.17 (4.68–5.64)	0.532
Sodium mmol/L	139 (137–141)	139 (137–140)	0.517
Glucose (mg/dL)	102.5 (88.8–127.85)	105.7 (92.0–170.8)	0.184
BUN (mg/dL)	129.8 (97.25–168.13)	119.8 (87.8–153.8)	0.058
Creatinine (mg/dL)	6.58 (5.0–8.32)	6.22 (5.27–7.55)	0.288
Hemoglobin g/dL	10.0 (8.59–11.35)	10.0 (8.54–11.4)	0.961
Hematocrit %	30.6 (26.6–35.0)	30.1 (27.15–35.1)	0.984
Neutrophils × 10^3^/μL	5.26 (3.97–6.54)	5.5 (4.34–6.91)	0.272
Lymphocytes × 10^3^/μL	1.4 (1.05–1.94)	1.45 (1.09–2.06)	0.395
Monocyte × 10^3^/μL	0.59 (0.45–0.76)	0.67 (0.52–0.84)	0.058
PLT × 10^3^/μL	211 (170.0–279.5)	221 (183.35–297.0)	0.119
Vascular Mapping Determinations *, median (Q1–Q3)			
Arterial Diameter (mm)	3.28 ± 1.14	2.95 ± 1.20	0.005
Vein Diameter (mm)	3.32 ± 0.93	2.97 ± 0.89	0.005
AVF Type and Placement, no. (%)			
RC-AVF	77 (46.11%)	51 (62.96%)	0.013
BC-AVF	65 (38.92%)	25 (30.86%)	0.216
BB-AVF	25 (14.97%)	5 (6.17%)	0.046
Non-Dominant Upper Limb	140 (83.83%)	68 (83.95%)	0.893
Outpatients, no. (%)	100 (59.88%)	39 (48.15%)	0.033
6-week Maturation Failure, no. (%)	12 (7.19%)	35 (43.21%)	<0.001
Dialysis on CVC at the time of AVF creation, no. (%)	74 (44.31%)	58 (71.60%)	<0.001
Follow-up period (years) mean ± SD	2.42 ± 1.46	2.02 ± 1.58	0.046

* The value of preoperative vascular mapping determinations is available only for a group of 212 patients from the entire cohort.

**Table 3:** Risk factors associated with long-term AVF failure.

Variables		AVF Failure		
		HR	95% CI	*p* Value
Female		1.92	1.24–2.97	0.003
Atrial Fibrillation		1.21	0.61–2.42	0.587
Diabetes		1.35	0.87–2.11	0.182
History of Myocardial Infarction		1.41	0.72–2.72	0.317
Active Smoking		1.89	1.15–3.09	0.011
AVF Type	RC-AVF	1.98	1.25–3.13	0.003
	BC-AVF	0.49	0.20–1.23	0.132
	BB-AVF	0.61	0.37–0.98	0.043
Preoperative Artery Diameter		0.73 *	0.55–0.97	0.029
Preoperative Vein Diameter		0.68 *	0.52–0.89	0.005

* Was calculated for patients with available preoperative artery and vein diameter data (n = 212).

**Table 4:** Multivariate analysis of dialysis on the CVC at the time of AVF creation on long-term AVF failure.

Variables		AVF Failure		
		HR	95% CI	*p* Value
Dialysis on the CVC at the time of AVF creation	Model 1	2.53	1.56–4.10	<0.001
	Model 2	2.86	1.75–4.68	<0.001
	Model 3	2.88	1.64–5.07	<0.001
	Model 4 *	3.13	1.72–5.69	<0.001

Model 1: unadjusted; Model 2: age and sex; Model 3: age, sex, and CV risk factors (diabetes, hypertension, ischemic heart disease, active smoking), and renal function (BUN, creatinine); Model 4: age, sex, CV risk factors (diabetes, hypertension, ischemic heart disease, active smoking), renal function (BUN, creatinine), AVF type, and preoperative vascular mapping. * The model was calculated for patients with available preoperative artery and vein diameter data (n = 212).

## Data Availability

Any data relevant to this case that are not presented in this manuscript can be obtained from the corresponding author upon reasonable request.

## Abbreviations

The following abbreviations are used in this manuscript:

AVF	Autologous arteriovenous fistula
VA	Vascular access
HD	Hemodialysis
AVG	Arteriovenous graft
CVC	Central venous catheter
ESVS	European Society of Vascular Surgery
CKD	Chronic kidney disease
ESKD	End-stage kidney disease
RC-AVF	Radiocephalic arteriovenous fistula
BC-AVF	Brachiocephalic arteriovenous fistula
BB-AVF	Brachiobasilic arteriovenous fistula
RA	Radial artery
BA	Brachial artery
BV	Basilic vein
CV	Cephalic vein
BUN	Blood urea nitrogen
SD	Standard deviation
